# Global analysis of human glycosyltransferases reveals novel targets for pancreatic cancer pathogenesis

**DOI:** 10.1038/s41416-020-0772-3

**Published:** 2020-03-19

**Authors:** Rohitesh Gupta, Frank Leon, Christopher M. Thompson, Ramakrishna Nimmakayala, Saswati Karmakar, Palanisamy Nallasamy, Seema Chugh, Dipakkumar R. Prajapati, Satyanarayana Rachagani, Sushil Kumar, Moorthy P. Ponnusamy

**Affiliations:** 1 Department of Biochemistry and Molecular Biology, University of Nebraska Medical Center, Omaha, NE USA; 2 Department of Pathology and Microbiology, University of Nebraska Medical Center, Omaha, NE USA; 3 Eppley Institute for Research in Cancer and Allied Diseases, Fred & Pamela Buffett Cancer Center, University of Nebraska Medical Center, Omaha, NE USA

**Keywords:** Tumour-suppressor proteins, Glycobiology, Glycobiology

## Abstract

**Background:**

Several reports have shown the role of glycosylation in pancreatic cancer (PC), but a global systematic screening of specific glycosyltransferases (glycoTs) in its progression remains unknown.

**Methods:**

We demonstrate a rigorous top-down approach using TCGA-based RNA-Seq analysis, multi-step validation using RT-qPCR, immunoblots and immunohistochemistry. We identified six unique glycoTs (B3GNT3, B4GALNT3, FUT3, FUT6, GCNT3 and MGAT3) in PC pathogenesis and studied their function using CRISPR/Cas9-based KD systems.

**Results:**

Serial metastatic in vitro models using T3M4 and HPAF/CD18, generated in house, exhibited decreases in B3GNT3, FUT3 and GCNT3 expression on increasing metastatic potential. Immunohistochemistry identified clinical significance for GCNT3, B4GALNT3 and MGAT3 in PC. Furthermore, the effects of B3GNT3, FUT3, GCNT3 and MGAT3 were shown on proliferation, migration, EMT and stem cell markers in CD18 cell line. Talniflumate, GCNT3 inhibitor, reduced colony formation and migration in T3M4 and CD18 cells. Moreover, we found that loss of GCNT3 suppresses PC progression and metastasis by downregulating cell cycle genes and β-catenin/MUC4 axis. For GCNT3, proteomics revealed downregulation of MUC5AC, MUC1, MUC5B including many other proteins.

**Conclusions:**

Collectively, we demonstrate a critical role of O- and N-linked glycoTs in PC progression and delineate the mechanism encompassing the role of GCNT3 in PC.

## Background

Pancreatic cancer (PC) is one of the most lethal cancers globally.^1^ While there are a multitude of domains, which contribute to the aggressiveness of this cancer, treatment options are limited. Various approaches have been investigated in an effort to address the poor survival rate of PC; however, substantial progress has yet to be achieved.^2^ This is mainly due to two factors: unavailability of a reliable diagnostic marker and poor mechanistic insights of disease pathogenesis. The discovery of CA19-9 as a diagnostic marker for PC has helped to address some of the challenges.^3–5^ CA19-9 is a sLe^a^-type glycan structure found either on cell membranes or on circulating secretory glycoproteins. The discovery of this marker towards the diagnosis of PC has helped in understanding the importance of glycogenes in PC.

Glycosylation is an ubiquitous post-translation modification (PTM) process that results in the addition of carbohydrates onto a variety of structures, including proteins.^6^ A multitude of membrane-bound or circulatory proteins are glycosylated. This PTM alters their biological functions. Among known glycosylation processes, O- and N-linked glycosylation are most commonly known to regulate protein functions on ser/thr motifs and asparagine residues, respectively. In cancerous conditions, physiological glycan-synthesis processes are disrupted leading to the synthesis of truncated Tn- (GalNAc), T- (Galβ1,3GalNAc) and Sialyl-Tn (Neu5Acα2,3GalNAc) structures on protein backbone.^7^ In addition, glycans involved in metastasis such as sLe^x^ and sLe^a^ are mostly upregulated driving the progression of PC.^8^ The above stated glycans are mostly O-linked whereas N-linked hybrid and complex glycans are increased leading to cancer progression.^7^ The synthesis of truncated and novel glycans during malignant conditions is governed by variety of glycosyltransferases. In this context, as there are more than 200 glycosyltransferases that act to regulate glycosylation, it is imperative to utilise an unbiased global approach to identify glycogenes involved in PC.^9^

In this study, we investigated the involvement of major glycosyltransferases in PC using a top-down approach. Using the TCGA RNA-Seq. dataset, PC cell lines, human and mouse tissues we identified the top-upregulated glycoTs in PC. Herein, KPC (LSL-Kras^G12D/+^, LSL-Trp53^R172H/+^, Pdx-1-Cre) mouse was used, which represents pancreatic ductal adenocarcinoma in mice. From this, we narrowed down to 6 glycoTs by comparing their transcriptional and translational levels in different in vitro and in vivo models. Following this identification, CRISPR-Cas9-mediated silencing of B3GNT3, GCNT3, FUT3 and MGAT3 was conducted, which resulted in distinct genotypic and phenotypic results in PC cell line HPAF/CD18. We identified the mechanism governing the action of GCNT3 in PC. Overall, this study attempts to evaluate key glycoTs participating in PC progression and metastasis.

## Methods

### Antibodies and reagents

Antibodies for B3GNT3, B3GNT6, B4GALNT3, FUT3, FUT6, MGAT3, ST6GalNAc-I, mucins, epithelial-mesenchymal transition (EMT) and stem cell markers were utilised herein (Supplemental Table I). Primers were designed for the variety of glycosyltransferases and were purchased from Eurofins Genomics LLC (Supplemental Table II). CRISPR/Cas9 sgRNA were purchased from Genscript (Supplemental Table III).

### Cell culture

Human pancreatic cancer cell lines (MIAPaCa-2, Capan-1, HPAF/CD18) were purchased from ATCC. HPNE was a kind gift from Dr. Michel Ouellete (Department of Internal Medicine, University of Nebraska Medical Center, Omaha, NE). MIAPaCa-2, Capan-1 and HPAF/CD18 were cultured in DMEM, High glucose supplemented with 10% FBS. HPNE was cultured in DMEM: M3 Base (3:1 volume ratio), 5% FBS, EGF.^10^ All the experiments were carried out using early passages of these cell lines. For CRISPR/Cas9 editing, HPAF/CD18 luciferase-positive cells were used and cultured under same conditions.

### TCGA RNA-Seq. data analysis

Normalised expression of all known glycosyltransferases (*n* = 207) was selected from RNA-Seq. data for TCGA pancreatic cancer subjects and stratified about the median proportion of malignant cells present in the sample as calculated using the ABSOLUTE purity score reported in TCGA (high cellularity, *n* = 74; low cellularity, *n* = 75) and adjacent normal sample (*n* = 3).^11^ The fold-change of each gene was calculated using the geometric mean between each paired group combination (high vs. low, high vs. normal, low vs. normal).

### In silico analysis of glycoTs in PC datasets

The Michigan portal (www.mipanda.org) and the gepia database (http://gepia.cancer-pku.cn/), which can be used for the analysis of next generations sequencing (NGS) data, was utilised. Herein, pancreatic samples specific data for B3GNT3, B3GNT6, B4GALNT3, GCNT3, FUT3, FUT6 and MGAT3 were obtained. The data were downloaded using the website and plotted henceforth.^12,13^

### IHC analysis

For antibody concentration optimisation, we obtained tissue slides from the tissue science facility. For this, the tissue sections (4 µm) were cut and mounted on super-frost, positively charged glass slides. Different antibody concentration was used to stain the tissues. The universal secondary antibody from Impress reagent kit (VECTOR, Burlingame, CA) were employed for mouse/rabbit generated antibody. For goat antibodies, anti-goat HRP was used. After incubation with the primary and secondary antibody and subsequent washing, the colour was developed with the peroxidase kit (VECTOR).

### Data analyses

For uniformity in scoring, each sample was given a composite score based on intensity and extent of tissue staining. Intensity was graded on a four-point scale: −, +, ++, and + ++. These values were given a numeric score – [0], +[1], ++[2] and + ++[3]. The extent of staining was graded on a four-point scale: 1 (0–24%), 2 (25–49%), 3 (50–74%) and 4 (75–100%). The composite score was obtained by multiplying the two values together and ranged from 0 to 12. The identity of histological stages of pancreatic cancer (PC) tissues and normal pancreas (NP) was determined by an independent pathologist at UNMC.^14^

### Genetically engineered KPC mice model

The present study was conducted in accordance with U.S. Public Health Service, “Guidelines for the care and use of laboratory animals”, ARRIVE guidelines and with approval of the UNMC Institutional Animal Care and Use Committee (IACUC). The mouse specimens used in the study were procured from the KPC and age-matched littermate control mice generated as per UNMC-IACUC-approved breeding (18-161-01-FC) and experimental (17-135-01-FC) protocols.^15^ The detailed explanation of the KPC mouse model and animal euthanasia is included as a method in the supplemental document (Supply2019.doc).

### RNA isolation, cDNA synthesis and qRT-PCR analysis

Total RNA was isolated using optimised and established protocols in our lab^15,16^ from human PC cell lines (HPNE, MIAPaCa-2, Capan-1 and HPAF/CD18, RNAeasy kit (Qiagen, CA, USA). Similar approaches were adapted to isolate total RNA (mirVana miRNA isolation kit (Thermo Fisher Scientific) from histologically confirmed human normal pancreas, pancreatic tumour (Tissue Bank UNMC- IRB186-14-EP) and KPC (LSL-Kras^G12D/+^, LSL-Trp53^R172H/+^, Pdx-1-Cre) C57BL/6 mice of either sex. Based on the tissues available at the time, we derived RNA from pancreatic tumours of 5 (*N* = 2) and 25 weeks (*N* = 3) of age along with normal pancreas (*N* = 3) from littermate control mice. Briefly, human cell lines, human and mouse tissues were extracted in RLT buffer in 1% β-mercaptoethanol, followed by cDNA synthesis using 2 μg of RNA for each sample. Quantitative-PCR (CFX Connect™ Real-Time system, Bio-Rad) was performed in 96-well plates with 1 μl of cDNA per sample with glycosyltransferases gene-specific primers and SYBR Green master mix reagent (Thermo Fisher Scientific). Gene expression data from qRT-PCR were collected, analysed and visualised using CFX Maestro Software accompanied by Real-Time PCR system. Data were double normalised based on β-actin expression and specific glycosyltransferases gene expression for respective control samples.

### Growth assay

For this, control and KD cells were cultured in DMEM (with 10% FBS) media and seeded at concentrations of 3000–4000 cells/well. After every 24 h for a total of 7 days, cells were incubated with 10 ul of MTT (5 mg/mL) in plain DMEM (90ul) for 3–4 h. Thereafter, cells were lysed by adding 200 ul of DMSO and incubated for 15–20 min. Absorbance was measured at 570 nm with the background reading calculated at 640 nm using ELISA plate reader.

### Immunoblots, mucin blots and lectin blots assay

HPNE, MIAPaCa-2, Capan-1 and HPAF/CD18 cells were lysed in RIPA buffer and protein was extracted from the lysates by freeze-thaw and syringe passage methods. Standard immunoblot conditions were used for running and transfer processes. For probing, following antibodies were used: anti-OCT3/4, anti-SOX2, anti-SOX9, anti-CD44, anti-b-catenin, anti-E-cadherin, anti-ZO1, anti-Zeb1, anti-Snail, anti-MUC4, anti-MALII, anti-B3GNT3, anti-B4GALNT3, anti-FUT3, anti-GCNT3, anti-FUT6, anti-MGAT3. β-actin was used for normalising immunoblots. Secondary antibody conjugated to HRP was used. For mucin blotting, 2% agarose gel was prepared and run on a horizontal gel for 4–5 h. Following electrophoresis, protein was transferred overnight by the capillary action. For lectin blotting, standard western blot techniques were used except that the blocking was performed in 3% BSA (Jackson Immunoresearch Labs, Inc.) and secondary incubation was performed in 3% BSA. Antibody used in this case where biotinylated (Vector Labs, CA, USA) and probed with streptavidin-HRP. In all cases, a chemiluminescent HRP kit was used (Bio-Rad, CA, USA).^17^

### CRISPR/Cas9 gene editing

Knockdown for B3GNT3, GCNT3, FUT3 and MGAT3 was performed using the CRISPR/Cas9 system in HPAF/CD18 cells. Briefly, cells were transfected with the gene-specific sgRNA ligated to CRISPR/Cas9 vector (pSpCas9 BB-@A-GFP, GenScript, NJ, USA) using either Turbofect or Polyplus reagents and following the manufacturer’s recommendation. After 48 h of transfection, GFP positive cells were sorted as either single or double cells and seeded into a 96-well plate. After 4–6 weeks, the clones were collected and checked for knockdown using immunoblot.^15,18^

### Colony formation assay

Cells were seeded in triplicates at concentration of 500–1000 cells/well in six-well plates. After 14–20 days of growth, the cells were fixed with ice-cold methanol for 20 min and stained with crystal violet stain solution (0.1% w/v in methanol). Colonies were counted using the “Find Maxima” function on ImageJ software available freely from NIH.^19^

### Wound-healing (scratch) assay

Cell migration was evaluated in B3GNT3, GCNT3, FUT3 and MGAT3 using the control and knockdown cells using a wound-healing assay as described previously.^20^ Wound closure was evaluated in the control and knockdown cells using ImageJ software.

### Mass spectrometry-based proteomics

The protocol for mass spectrometry-based proteomics is expanded in the supplemental file.

### Statistical analysis

ANOVA and/or Student’s *t*-test was used to determine the statistical rigor in all the experiments. A *p*-value of ≤0.05 was considered statistically significant.

## Results

### Top-down transcriptomic analysis reveals overexpression of B3GNT3, B4GALNT3, FUT3, FUT6, GCNT3 and MGAT3 in PC

**Fig. 1:**
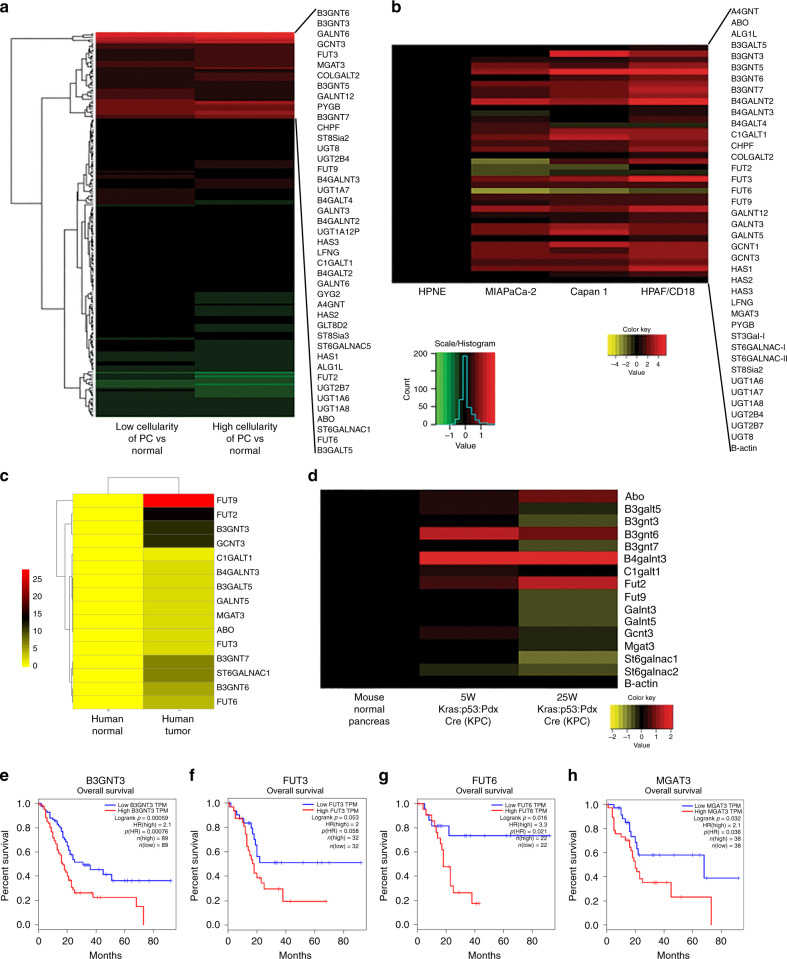
Transcriptomic analysis of glycoTs. **a** Heatmap based on the RNAseq analysis for more than 200 glycoTs in high cellularity (tumour), low cellularity (tumour) and normal patients as obtained from the TCGA datasets. **b** RT-qPCR analysis for 39 glycoTs in HPNE, MIAPaCa-2, Capan-1 and HPAF/CD18 cell lines. **c** RT-qPCR analysis for the 17 glycoTs in human tissues based on normal pancreas (N = 3) and pancreatic tumour (*N* = 3). **d** RT-qPCR for mouse glycoTs in the normal animal, 5-week-old KPC and 25-week-old KPC animal. **e**–**h** Survival plots were obtained from the gepia dataset and reported for B3GNT3, FUT3, FUT6 and MGAT3.

There are more than 200 glycosyltransferases that facilitate the enzymatic addition of a variety of carbohydrate structures in the human cell.^21^ In this study, the variable expression of these enzymes in pancreatic cancer was evaluated using different systems. Initially, analysis of the expression of all the glycosyltransferases from the RNA-Seq. data in the TCGA dataset were conducted. For this, the cases were classified as high cellularity (tumour), low cellularity (tumour) and normal patient samples. Some of the glycoTs genes such as *GCNT3, GALNT5, FUT3, B3GNT6* and *B3GNT3* were more than 19-fold overexpressed in tumour samples. For further analysis in cells and tissues, we considered all those enzymes that were more than 2-fold overexpressed in tumourous samples (Fig. 1a). GlycoTs genes overexpressed are as follows: *A4GNT, ABO, ALG1L, B3GALT5, B3GNT3, B3GNT5, B3GNT6, B3GNT7, B4GALNT2, B4GALNT3, B4GALT4, C1GALT1, CHPF, COLGALT2, FUT2, FUT3, FUT6, FUT9, GALNT12, GALNT3, GALNT5,GCNT3, HAS1, HAS2, HAS3, LFNG, MGAT3, PYGB, ST6GALNAC1, ST8SIA2, UGT1A6, UGT1A7, UGT1A8, UGT2B4, UGT2B7* and *UGT8*. In addition, ST3GAL1, ST6GALNAC2 and GCNT1 were also screened due to their important role in O-glycosylation.

Next, we selected these genes for further analysis by RT-qPCR in HPNE, MIAPaCa-2, Capan-1 and HPAF/CD18 cell lines. Among the noted glycoTs, 22 genes were found to be significantly upregulated in aggressive PC cell lines Capan-1 and HPAF/CD18. The significantly overexpressed genes are *ABO, B3GALT5, B3GNT3, B3GNT6, B3GNT7, B4GALNT2, B4GALNT3, FUT2, FUT3, FUT6, FUT9, GALNT3, GALNT5, GCNT3, MGAT3, ST6GALNAC1, ST6GALNAC2, UGT1A6, UGT1A7, UGT1A8, UGT2B4* and *UGT2B7* (Fig. 1b). Based on the results from Capan-1 and HPAF/CD18, we selected the top 20 upregulated genes identified for analysis in other in vitro and PC tissue-based models. A PC progression model was used, wherein HPNE cell lines were induced with E6E7, E6E7st, E6E7KRas and E6E7stKRas, respectively. In this in vitro progression model, HPNE cells become aggressive following the introduction of KRas mutation along with other oncogenes.^22^ We found that many genes including *B3GNT3, B4GALNT3, FUT3, FUT6, GCNT3* and *MGAT3* were elevated significantly in the HPNE E6E7stKRas sample as compared to the HPNE control. In addition, for some of these genes, the fold-change values increased progressively on introduction of KRas (Fig. S1).

Based on the above results, relative expression of top-regulated O-linked and N-linked glycogenes were identified in human and mouse PC tissues. In human tissues, many genes including *B3GNT3, B4GALNT3, FUT6, FUT9* and *GCNT3* were elevated (Fig. 1c). Homologous genes for these glycoTs were identified in mouse. The list is as follows: *Abo, B3galt5, B3gnt3, B3gnt6, B3gnt7, B4galnt2, B4galnt3, Fut2, Fut9, Galnt3, Galnt5, Gcnt3, Mgat3, St6galnac1* and *St6galnac2*. Pancreatic tissues from (LSL-Kras^G12D/+^, LSL-Trp53^R172H/+^, Pdx-1-Cre) KPC mouse at the age of 5 and 25 weeks were isolated. These tissues were compared with the pancreas from normal mouse. We consistently found that as compared to the normal pancreatic tissue, *Abo, B3gnt6, B4galnt3* and *Fut2* were highly upregulated in mouse PC tissues (Fig. 1d). In contrast to mouse tissues, ABO and FUT2 were not differentially expressed in human cell lines or tissues. Based on the above findings, we selected 6 genes (*B3GNT3, B4GALNT3, FUT3, FUT6, GCNT3* and *MGAT3)* for further analysis (Table SIV). We found that B3GNT3, FUT3, FUT6 and MGAT3 exhibited poor-overall survival in pancreatic patient samples as reported on the gepia dataset (Fig. 1e–h). Moreover, the relative expression of these six genes was also evaluated using mipanda, gepia and cbioportal databases (Figs. S2, S3). In coherence with the RT-PCR data, all of these genes demonstrated elevated expression in tumour samples compared to the normal. In addition, FPKM values were also reported for all these six genes based on the TCGA analysis that showed increased expression in high and low cellularity samples versus normal (Figure S3B–H).

### Protein expression profiling reveals differential role for B3GNT3, B4GALNT3, FUT3, FUT6, GCNT3 and MGAT3 in metastatic PC cell lines

**Fig. 2:**
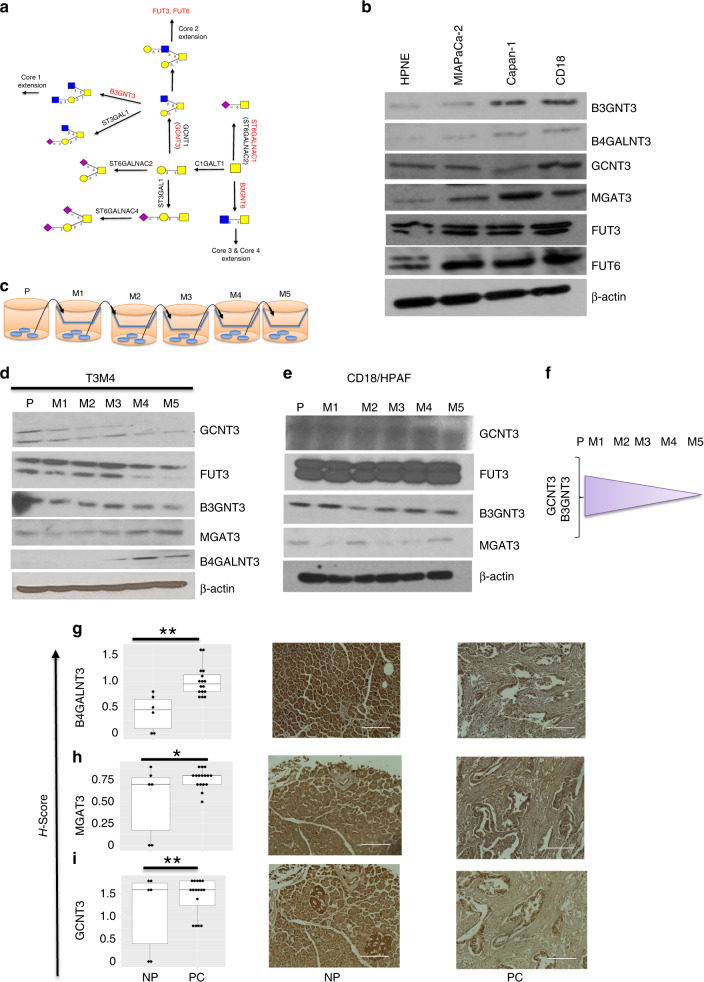
Translational and IHC based analysis to probe glycoTs. **a** Schematic showing the progression of O-glycosylation in human systems. **b** Immunoblots for the selective glycoTs B3GNT3, B4GALNT3, FUT3, FUT6, GCNT3 and MGAT3 in the HPNE, MIAPaCa-2, Capan1 and HPAF/CD18 cell lines. **c** Schematic showing the generation of metastatic cell lines from P to M5. **d**, **e** Immunoblots for the above stated glycoTs in the metastatic cell lines of T3M4 and HPAF/CD18. **f** Schematic illustrating the changes in the expression of glycoTs from P to M5. **g**–**i** Immunohistochemistry analysis for B4GALNT3, MGAT3 and GCNT3 in normal pancreas and pancreatic cancer tissues. Representative images corresponding to the normal pancreas and pancreatic adenocarcinoma tissues are mentioned. Details of the approach used is mentioned in methods section. * indicates *p* < 0.05 and ** indicates *p* < 0.005 in all the cases.

All the six glycogenes identified above were further evaluated at protein level in HPNE, MIAPaCa-2, Capan-1 and HPAF/CD18 cell lines. Some of the genes studied in the current work are as presented in the cartoon for O-glycan extension (Fig. 2a). We found higher protein levels for all these genes, namely B3GNT3, B4GALNT3, GCNT3, MGAT3, FUT3 and FUT6 in either capan-1 or HPAF/CD18 or both (Fig. 2b). In order to study the role of these glycoTs in metastasis, we systematically developed metastatic sub-lines for T3M4 and HPAF/CD18. These sub-lines were generated by collecting cells that migrated from the trans well chamber after time point of 24 h (M1), 12 h (M2), 12 h (M3), 6 h (M4) and 3 h (M5) (Fig. 2c). Herein, levels of GCNT3 and B3GNT3 decreased from P to M5 in both T3M4 and HPAF/CD18 cell lines. FUT3 level decreased only in T3M4 cell line. Similarly, MGAT3 participated in metastasis in T3M4 and HPAF/CD18 cell lines whereas increased expression of B4GALNT3 was observed only in T3M4 cell line (Fig. 2d–f).

### Immunohistochemistry reveals increased staining for GCNT3, MGAT3 and B4GALNT3 in human pancreatic tissue microarray

To further evaluate the clinical significance of these glycoTs, we conducted immunohistochemistry for B3GNT3, B4GALNT3, FUT3, GCNT3 and MGAT3 on human pancreatic tissue microarrays. Pathologically, the tumours were comprised of ductal adenocarcinoma, adenosquamous carcinoma and neuroendocrine carcinomas (Males = 12, Females = 4). Also, these tumour tissues were comprised of AJCC stages IIA and IIB. We found the acinar cells stained more positive for all the glycoTs when compared to the ductal cells in normal tissues. B3GNT3 had higher (*p* > 0.05) and FUT3 had equivalent (*p* > 0.05) expression in the ductal compartments of tumour tissues as compared to the control tissues (Fig. S4A, B). We found that the expression of B4GALNT3 (*p* = 0.0003) and MGAT3 (p = 0.01) was significantly higher in PC as compared to normal pancreas (Fig. 2g, h). For GCNT3, the mean H-score was higher in PC as compared to normal pancreas and this difference was found to be significant through ANOVA (*p* = 0.008) (Fig. 2i).

### B3GNT3 inhibits malignancy whereas FUT3, GCNT3 and MGAT3 induces it in PC cells

**Fig. 3:**
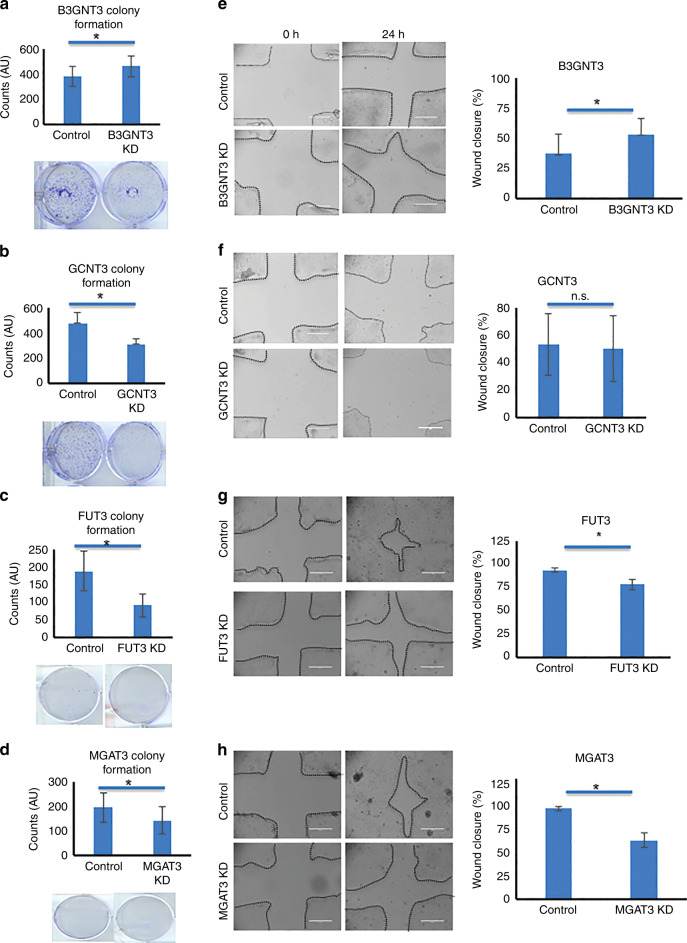
Functional analysis of B3GNT3, GCNT3, FUT3 and MGAT3 in HPAF/CD18 cells. **a**–**d** Colony formation assays were conducted on the CRISPR/Cas9-edited glycoTs corresponding to B3GNT3 (a), GCNT3 (**b**), FUT3 (**c**) and MGAT3 (**d**). 500–1000 cells/ well were seeded at day 1 and allowed to culture in 2% FBS medium for a period of 14–20 days. At the end of this, cells were methanol fixed and stained to count. ImageJ was used for the quantification of these colonies. Details of the approach are mentioned in the methods section. Wherever lesser number of colonies with smaller size were present, it was relatively difficult to photography colonies. Here, AU indicates Arbitrary Unit as the colonies were counted using automated ImageJ method. **e**, **h** Wound closure was demonstrated in the presence and absence of B3GNT3 (**e**), GCNT3 (**f**), FUT3 (**g**) and MGAT3 (**h**). The bar graphs show quantification of the percentage wound closure as obtained for the control and CRISPR/Cas9-edited CD18/HPAF cells. * indicates p < 0.05 in all the cases.

To evaluate the functional consequences of increased glycoTs expression, we performed CRISPR/Cas9-based gene editing for 6 glycoTs in HPAF/CD18 cell line. Amongst these, B3GNT3, GCNT3, FUT3 and MGAT3 showed successful KD using the CRISPR/Cas9 constructs (Fig. S5A–D). Colony formation assays were conducted using these four cell systems. Significantly different effects were observed in all cases. B3GNT3 knockdown lead to increased colony formations compared to control cells (Fig. 3a). Knockdown of GCNT3 lead to a 20% reduction in colonies compared to the control cells (Fig. 3b).^23^ Significantly, fewer colonies were formed in FUT3 and MGAT3 knockdown cells (Fig. 3c, d). To determine the individual roles that glycoTs impart on cancer cell motility, wound-healing assay was conducted for all the CRISPR/Cas9 constructs. The ability to repair wounds was significantly increased in B3GNT3 knockdown cells compared to the control cells at 24-h timepoint (Fig. 3e). GCNT3 knockdown decreased wound closure at 24-h timepoint but this difference was found to be statistically insignificant (Fig. 3f). Similarly, knockdown of both FUT3 and MGAT3 showed decrease in wound closure at 24-h timepoint (Fig. 3g, h). Overall, these findings suggest that B3GNT3 inhibits PC and the remaining three genes promotes it.

### B3GNT3, FUT3 and MGAT3 knockdown has independent oncogenic regulators

**Fig. 4:**
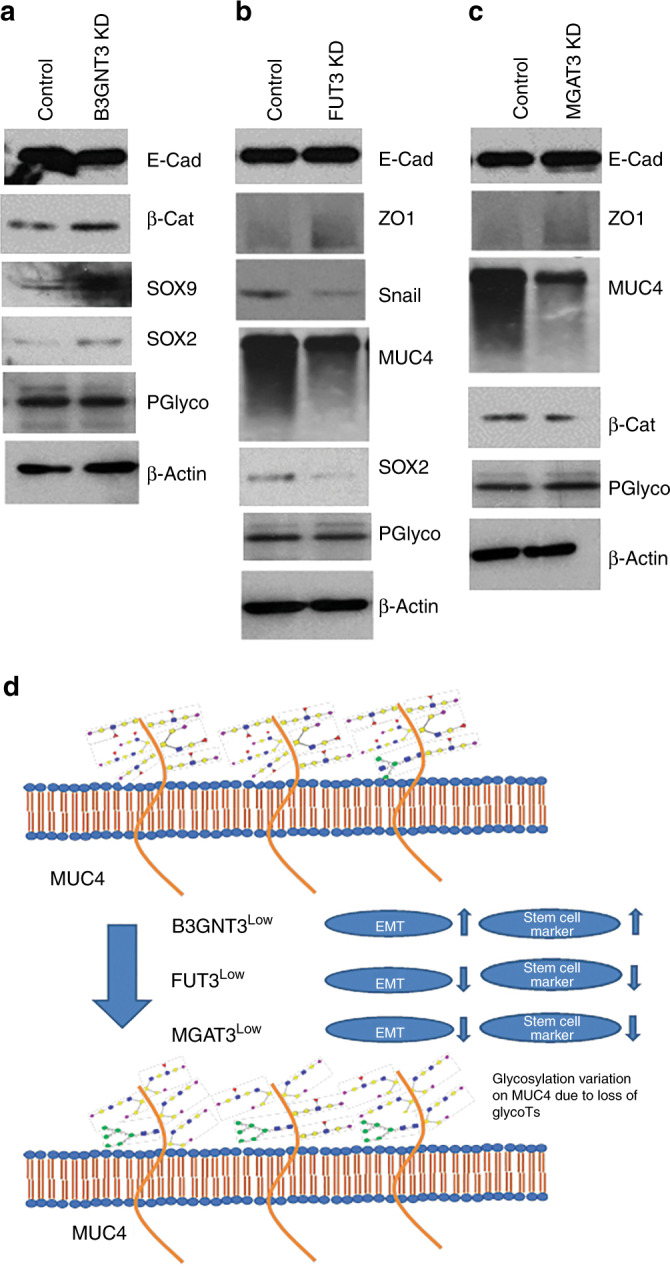
Effect on oncogenic pathways due to loss of B3GNT3, FUT3 and MGAT3. Mucins, EMT and stem cell markers were studied at protein level in the control and CRISPR/Cas9-edited HPAF/CD18 cells for B3GNT3 (**a**), FUT3 (**b**) and MGAT3 (**c**). The data illustrate role for these glycoTs on PC progression based on their ability to drive independent EMT and stem cell markers. **d** Schematic illustrating the role of B3GNT3, FUT3 and MGAT3 on mucin glycosylation, EMT and stem cell markers in the context of PC pathogenesis.

In order to explore the mechanisms governing proliferation capacity and motility of these KD systems, various oncogenes were investigated. We observed decreased expression of E-cadherin and increased expression of β-catenin in B3GNT3 knockdown cells. For both FUT3 and MGAT3 knockdown cells, there was higher expression of E-cadherin and ZO-1. In FUT3 knockdown cells, we also found lower expression of Snail, suggesting an influence by multiple EMT driving factors on motility (Fig. 4a–c).^24–26^ As EMT is associated with the progression and metastasis of tumour, the genotypic findings adhere with the phenotypic results obtained above. We have established the role of MUC4, a high molecular weight mucinous glycoprotein, in motility, invasion and metastasis of pancreatic cancer, thus we focused on MUC4 in the current study.^27^ Its expression was reduced significantly in FUT3 and MGAT3 KD cells, whereas B3GNT3 KD showed no change in MUC4 protein expression (Fig. 4a–c, S5 E). Since, stem cell markers are identified as one of the regulatory elements that participate in tumorigenicity,^28,29^ the role of these glycoTs on such genes was assessed. To this end, B3GNT3 knockdown elevated the expression of SOX2 and SOX9 (Fig. 4a). Also, there was elevated expression of Oct3/4, Integrin β1 and Integrin β5 (data not shown). This supported the increase in proliferation and motility observed in B3GNT3 KD cells. FUT3 KD reduced the expression of SOX2 (Fig. 4b) whereas β-catenin was reduced in MGAT3 KD cells (Fig. 4c). P glycoprotein (MDR1) levels were similar across all the KD cell lines. Based on the findings for B3GNT3, FUT3 and MGAT3 a schema illustrating their function is presented. This explains in a concise way, roles played by these genes in regulating MUC4 expression and other oncogenes (Fig. 4d).

### Talniflumate, GCNT3 inhibitor, reduces growth and migration in T3M4 and CD18 cell lines

**Fig. 5:**
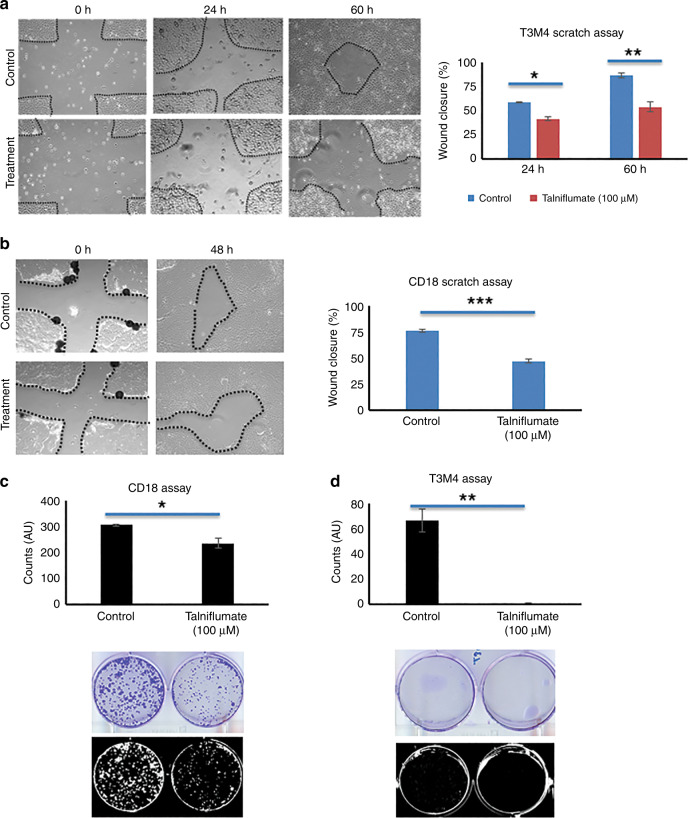
Talniflumate, GCNT3 inhibitor, was used at the concentration of 100uM and treated along with the DMSO control to T3M4 and CD18 cells. **a**–**b** Wound-healing results generated for the T3M4 and CD18 cells at different time points (*N* = 3). **c**–**d** Colony formation assay was conducted for T3M4 and CD18 cells by seeding 500–1000 cells/well and culturing in low medium condition to identify tumorigenicity potential. Images were taken after 14–20 days depending on the colonies formed in different cell systems (*N* = 3). * indicates *p* < 0.05, ** indicates *p* < 0.001 and *** indicates *p* < .0001 in all the cases. Wherever lesser number of colonies with smaller size were present, it was relatively difficult to photography colonies. Here, AU indicates Arbitrary Unit as the colonies were counted using automated ImageJ method.

Rao et al. identified an inhibitor, Talniflumate, affecting the expressions of GCNT3 in pancreatic cancer pathogenesis. We used this inhibitor in the current study to elucidate its function in distinct pancreatic cancer cell lines. To this end, we identified the expression of GCNT3 in T3M4 as compared to HPNE and found its expression to be higher in T3M4^30^ (Figure S5F). For both T3M4 and CD18 cell lines, we observed significantly reduced colony formation and migration in talniflumate treated samples as compared to the DMSO control. The concentration of talniflumate used was 100 uM. This analysis further affirmed that GCNT3 loss indeed reduces the proliferation and migratory capacity of PC (Fig. 5a–d).

### Loss of GCNT3 suppresses tumorigenesis by regulating β-catenin/MUC4 axis in PC cells

**Fig. 6:**
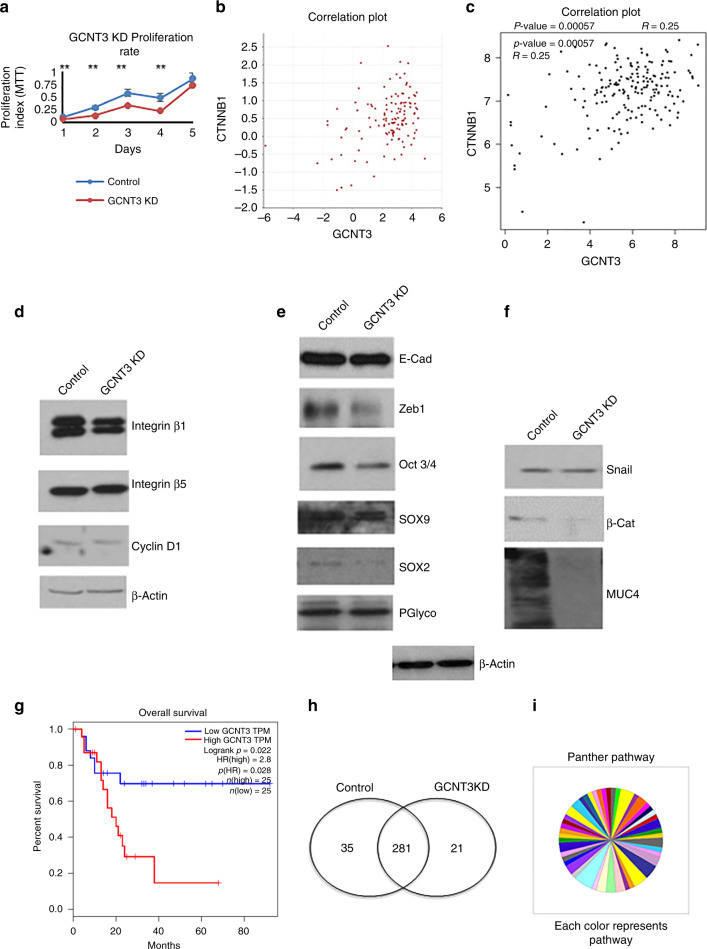
Effect of loss of GCNT3 on oncogenic pathways. **a** Cell proliferation rate studied for the control and GCNT3 KD cells using MTT assay at different days. **b**, **c** Correlation plot obtained for GCNT3 and β-catenin using R2 and gepia database. In both the cases, significant correlation with *p* < 0.05 was obtained. **d** Western blot showing changes in genes involved in cell cycle for the control and GCNT3 KD. **e**, **f** Western blot for the EMT, stem cell markers and β-catenin/MUC4 axis shown to support the proposition that GCNT3-dependent tumour suppression is b-catenin/MUC4 dependent. **g** Survival plot for the GCNT3 obtained using gepia database showing poor-overall survival with higher GCNT3 expression. **h** Venn diagram showing the proteins found in the control and GCNT3 KD system and those that were common to both the cell systems. **i** Panther based pathway analysis using the significantly downregulated proteins in the GCNT3 KD with respect to the control set.

We observed reduced growth profile for GCNT3 KD cells as compared to the control when studied consecutively for 5 days. This difference in the growth profile was found to be significant and thus confirmed that loss of GCNT3 reduces cell proliferation (Fig. 6a). We even observed that the expression of integrin β1, β5^31^ and cyclin D1 reduced in GCNT3 KD cells as compared to the control (Fig. 6d). Downregulation of these genes adhered with the results obtained for the growth and colony formation assay. We next studied EMT and stem cell markers to further explore the function of GCNT3. We found no change in E-cadherin but decrease in Zeb-1. Also, various stem cell markers such as Oct 3/4, SOX2, SOX9 were downregulated suggesting suppression of tumorigenic behaviour (Fig. 6e). We next evaluated MUC4 expression and found that loss of GCNT3 highly reduced the binding by 8G7 antibody on MUC4. To this end, we looked at the upstream molecules of MUC4 and found that Snail and β-catenin levels are reduced in GCNT3 KD cells^32^ (Fig. 6f, S5G). Also, GCNT3 significantly correlated with β-catenin as found using R2 and gepia database (Fig. 6b, c). This analysis further confirms their co-expression and regulation of β-catenin by GCNT3. Thus, we see that GCNT3 with poor-overall survival in PC pathogenesis regulates it by participating in various pathways including cell cycle markers, stem cell markers and β-catenin/MUC4 axis. (Fig. 6g).

### Proteomics reveals mechanistic insights due to the loss of GCNT3

To further delineate the mechanism governing the loss of GCNT3 in PDACs, we performed mass spectrometry-based proteomics analysis. To this end, whole-cell proteomics was performed on the control CD18 cells as compared to the GCNT3KD. We found majority of proteins associated with the control and GCNT3KD samples and about similar proteins specific to either samples (Fig. 6h). Using panther, we identified that various pathways including DNA replication, O-antigen synthesis, Integrin signalling, N-acetylglucosamine metabolism etc. were downregulated (Fig. 6i). Complete list of pathways downregulated in GCNT3KD is reported as Table S4. MUC5AC (*p* < 0.05) and MUC5B (*p* < 0.05) were significantly downregulated in GCNT3KD sample including many other proteins. Also, we found that CTNNB1P1 protein, which is the β-catenin interaction protein and inhibits wnt/β-catenin signalling was upregulated in GCNT3KD. This implicates that GCNT3 mediates PDAC progression and metastasis by regulating the β-catenin/MUC4 axis. More mechanistic insights on this aspect is part of our future studies and will be explored in greater depth later.

## Discussion

Pancreatic cancer initiation requires alterations in several biological factors including epigenetics, transcriptomics, metabolomics and post-translational modification.^1,2,33–35^ A variety of studies has focused on the role of post-translational modifications in pancreatic cancer. While these studies have shown the effects of O- and N-glycosylated genes in the progression of pancreatic cancer, a global analysis using an unbiased approach for targeting glycoTs is still missing. In the current study, we followed a top-down approach to identify key glycoTs upregulated in pancreatic cancer using TCGA RNA-Seq. data. After the multi-step validation, we identified B3GNT3, B4GALNT3, FUT3, FUT6, GCNT3 and MGAT3 as top-upregulated genes in aggressive pancreatic cancer cell lines Capan-1 and HPAF/CD18. Among these, we successfully identified the functions of B3GNT3, GCNT3, FUT3 and MGAT3 in pancreatic cancer cells.

We have previously shown that the loss of C1GALT1, which is an O-glycan branching enzyme, contributes to PC aggressiveness using a KPC and KPCC mouse model. This gene participates in the formation of both Core-1 and Core-2 branches on O-glycan and thus is an important post-translational regulatory element.^36,37^ In this study, we identified B3GNT3 and GCNT3 as two of the top-upregulated genes involved in the extension of Core-1 and Core-2 glycans, respectively. While B3GNT3 primarily adds GlcNAc to galactose, resulting in the extended Core-1 glycans, GCNT3 forms GlcNAc in the β1,6 linkage to GalNAc resulting in a trisaccharide Core-2 glycan.^38–40^ In addition, B3GNT3 also results in an extension of polylactosamine structures on Core-2 O-glycan.^41,42^ Various studies have shown that B3GNT3 is involved in the progression of colon, pancreatic, hepatocellular cancers, among others.^42,43^ In contrast, loss of B3GNT3 has been shown to result in increased migration and invasion in neuroblastoma cells.^44^ In coherence to the observations in neuroblastoma cells, the current study finds that loss of B3GNT3 results in increased proliferation and invasion. This suggests that B3GNT3 may behave differently when expressed in malignant cells of different cancers. In addition, we found an increase in the stem cell markers Oct3/4, SOX2 and SOX9 in the loss of B3GNT3 which suggests that this glycoT negatively regulates stem cell markers in HPAF/CD18 cells.^18,45^ In contrast, an earlier study from our group on B3GNT3 showed its role in the maintenance and self-renewal of pancreatic cancer stem cells.^46^ The mechanistic insights leading to these differences for B3GNT3 are current unknown. Furthermore, since acceptor specificity and reaction kinetic analysis for B3GNT3 is not known, we believe that its loss will result in an exposure of Galβ1,3GalNAc residues on the glycoproteins thus making the action by GCNT1 and GCNT3 more catalytically favourable.^36^ This contributes to higher sLex synthesis and increased tumour invasion and metastasis (Fig. S5E).^8,47^ Thus, the findings in our study are in accord with the traditional understanding of the role that B3GNT3 may play in regulating cancer cell migration and metastasis. While B3GNT3 loss increased invasion, GCNT3 loss reduced invasion. Downregulation of GCNT3 has been earlier shown to reduce invasion and migration in non-small cell lung cancer.^48^ Also, Rao et al. showed higher incidence of GCNT3 in human pancreatic tissues and 10-month-old Kras-induced genetically engineering mouse model.^30^ Higher expression of GCNT3 was associated with poor survival. The same group further identified talniflumate as an inhibitor of GCNT3, which also reduced mucin expression. In the current study, we also reported reduced colony and wound healing on treatment of talniflumate. Additionally, we report mechanism of GCNT3 regulation of PC by looking at various oncogenes forming part of EMT and stem cell. We also found that β-catenin/MUC4 axis acts as the regulatory factor in PC pathogenesis. An earlier study from our group has shown regulation of MUC4 by β-catenin and this axis was shown to be affected in the current study. To further delineate the mechanism, we performed proteomics in GCNT3 KD cells and found that mucins are downregulated. Additionally, proteomics analysis reported upregulation of CTNNBIP1 gene, which negatively regulates wnt/β-catenin signalling. Since other studies have not shown an effect of GCNT3 on EMT, stem cell and β-catenin/MUC4 axis, our study is the first to propose a possible mechanism by which GCNT3 may regulate PC.

FUT3 participates in O-linked glycan synthesis whereas MGAT3 is an N-linked glycoenzyme.^49,50^ FUT3 adds the terminal sugar fucose by α1,3 and α1,4 linkage resulting in the formation of sLe^x^ and sLe^a^ type glycan.^51–53^ Loss of FUT3 has been implicated in reducing tumour metastases and progression in a variety of cancers.^54–56^ Zhan et al. found that knockdown of FUT3 in Capan-1 cells decreased proliferation and migration. Furthermore, FUT3 KD inhibited EMT markers and suppressed tumour formation in orthotopically implanted animals.^57^ Similarly, in the current study, we observed reduced proliferation and migration in FUT3KD cells. In addition to the reduction in EMT markers, we also observed loss of SOX2 expression.^58^ This reduction in stem cell markers indicates that the loss of FUT3 may reduce metastatic burden.

MGAT3 is a bisecting enzyme that adds GlcNAc onto the mannosylated N-glycan. MGAT3 has been implicated in various cancers due to the important role it plays in reducing the hybrid and complex structures on N-glycans. MGAT3 participates in the developmental process as exhibited in an animal model. Loss of MGAT3 retards the liver tumour burden when induced using diethylnitrosamine.^59,60^ Moreover, higher levels of MGAT3 has been observed in metastatic colorectal cancer cell lines.^61^ However, in breast cancer and hepatocarcinoma, MGAT3 loss has been shown to increase proliferation and invasion.^62–65^ Thus, MGAT3 plays a cancer-dependent promotor or suppressor role. In the current study, we found inhibition in proliferation and invasion on loss of MGAT3 expression. EMT markers were also downregulated as compared to the control and there was a decrease in β-catenin levels in MGAT3 KD cells, implicating its role in the observed phenotypic differences.

Altogether, we have systematically analysed the role of B3GNT3, MGAT3, FUT3 and GCNT3 in pancreatic cancer and illustrated the mechanism of GCNT3 in PC. We believe this study will provide impetus to carry out further analysis in delineating the mechanisms governing PC. We plan to independently implant orthotopically these KD cell systems and understand the impact these glycoTs may have on the tumour progression and metastasis in PC.

## Supplementary information

Supplemental Material

Supplemental document

## Data Availability

Data generated during this study are included in this article and its supplementary files.
