# Correction: Global analysis of human glycosyltransferases reveals novel targets for pancreatic cancer pathogenesis

**DOI:** 10.1038/s41416-020-0842-6

**Published:** 2020-04-14

**Authors:** Rohitesh Gupta, Frank Leon, Christopher M. Thompson, Ramakrishna Nimmakayala, Saswati Karmakar, Palanisamy Nallasamy, Seema Chugh, Dipakkumar R. Prajapati, Satyanarayana Rachagani, Sushil Kumar, Moorthy P. Ponnusamy

**Affiliations:** 1 Department of Biochemistry and Molecular Biology, University of Nebraska Medical Center, Omaha, NE USA; 2 Department of Pathology and Microbiology, University of Nebraska Medical Center, Omaha, NE USA; 3 Eppley Institute for Research in Cancer and Allied Diseases, Fred & Pamela Buffett Cancer Center, University of Nebraska Medical Center, Omaha, NE USA

**Keywords:** Tumour-suppressor proteins, Glycobiology, Glycobiology

Correction to: *British Journal of Cancer* (2020) 10.1038/s41416-020-0772-3, published online 19 March 2020

Since the publication of this paper, the authors have noticed an error in the listed department of author Dipakkumar R. Prajapati, where the Department of Pathology and Microbiology was incorrectly listed as the Department of Pathology and Molecular Biology. This has been corrected below.

Dipakkumar R. Prajapati^2^

^2^Department of Pathology and Microbiology, University of Nebraska Medical Center, Omaha, NE, USA

