# Supplementary material for: Global analysis of human glycosyltransferases reveals novel targets for pancreatic cancer pathogenesis
Source: Br J Cancer. 2020 Mar 19;122(11):1661–72. doi: 10.1038/s41416-020-0772-3 (PMC7251111; doi:10.1038/s41416-020-0772-3)
Supplement: Supplementary file 1 — Supplemental Material [file 41416_2020_772_MOESM1_ESM.pptx]

## Slide 1
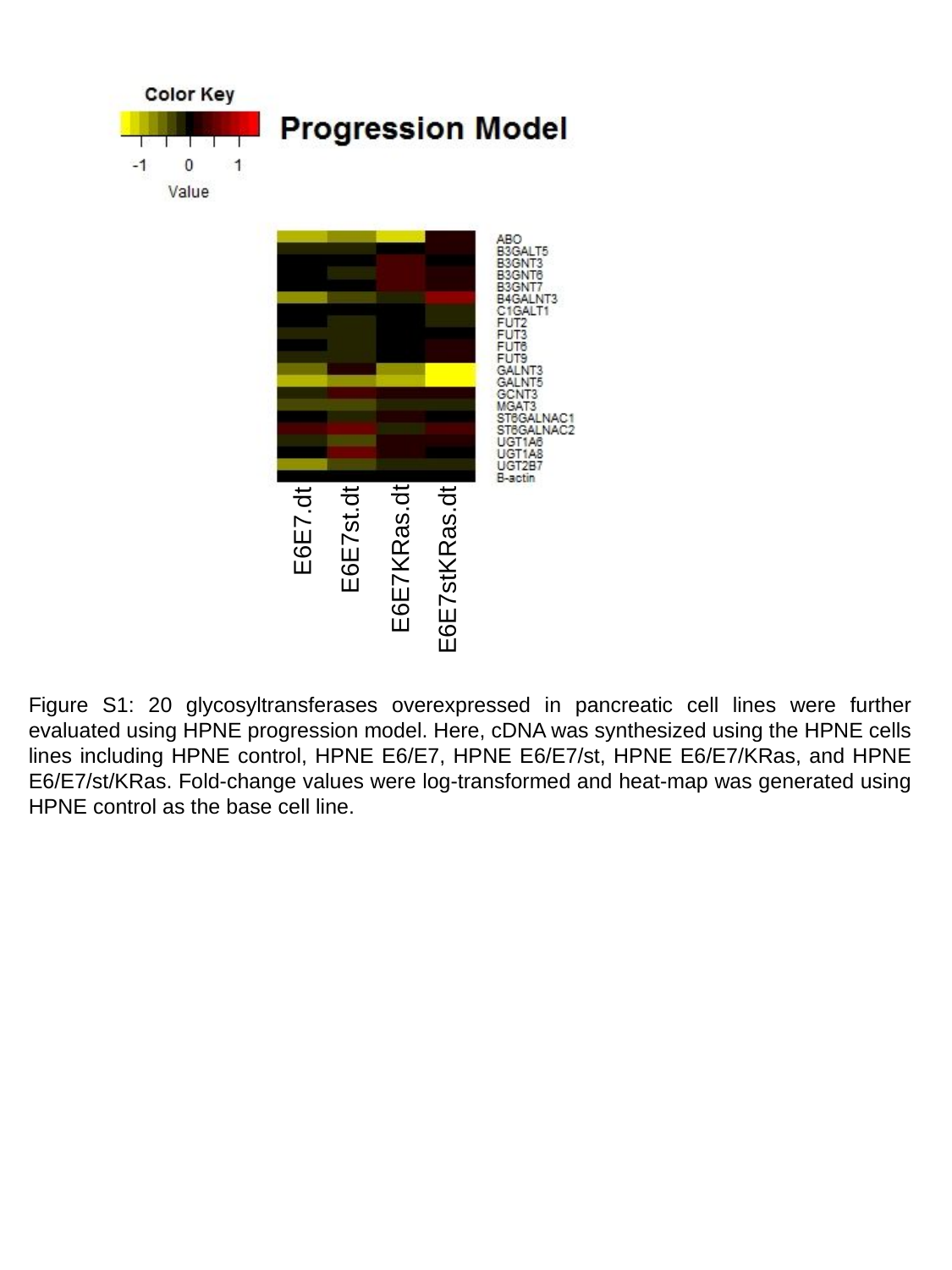

E6E7.dt
E6E7st.dt
E6E7KRas.dt
E6E7stKRas.dt
Figure S1: 20 glycosyltransferases overexpressed in pancreatic cell lines were further evaluated using HPNE progression model. Here, cDNA was synthesized using the HPNE cells lines including HPNE control, HPNE E6/E7, HPNE E6/E7/st, HPNE E6/E7/KRas, and HPNE E6/E7/st/KRas. Fold-change values were log-transformed and heat-map was generated using HPNE control as the base cell line.

## Slide 2
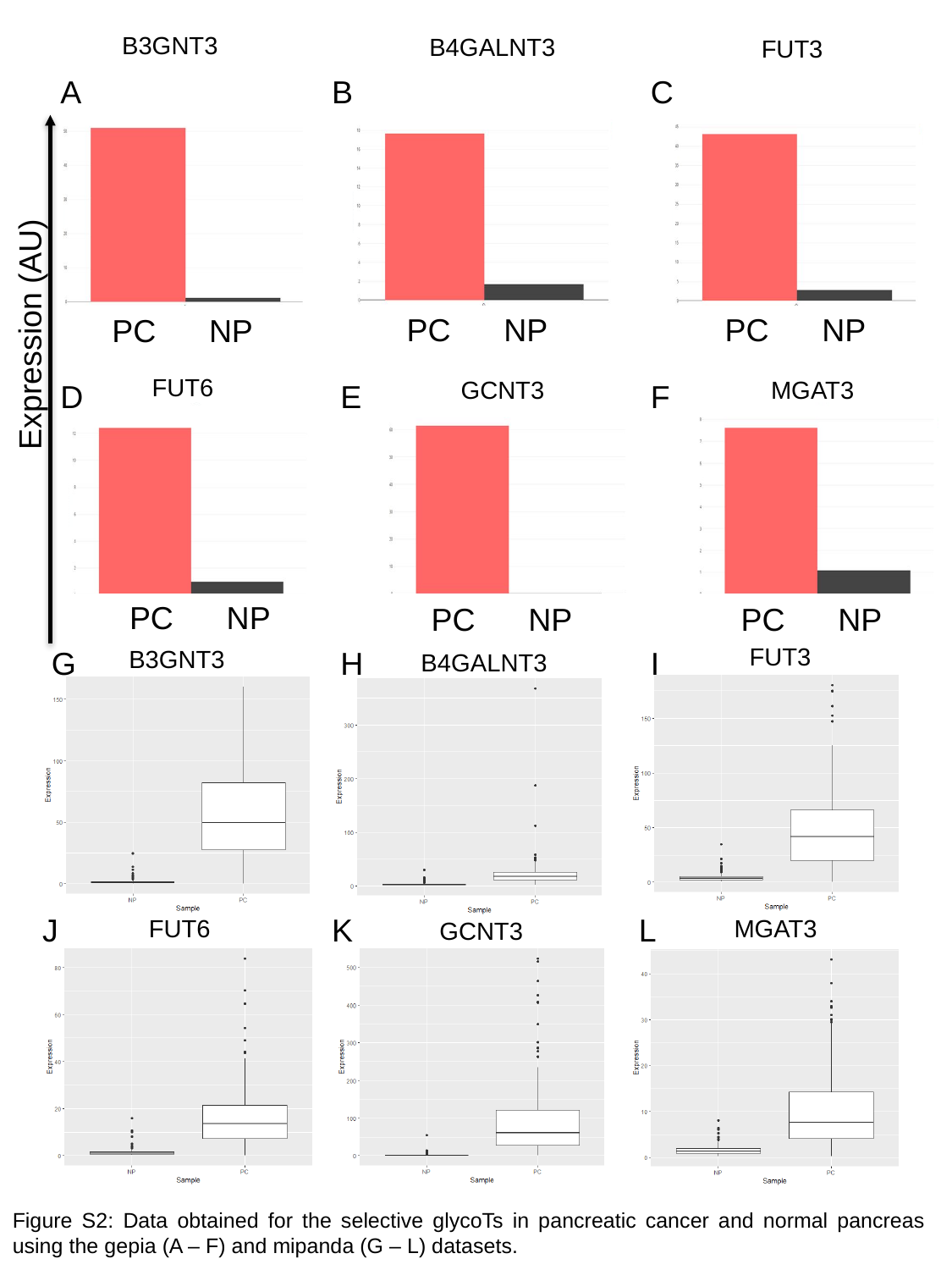

B3GNT3
B4GALNT3
FUT3
 A		 B			C
 D		 E			F
 G		 H			I
 J		 K		 L
Expression (AU)
PC NP
PC NP
PC NP
FUT6
GCNT3
MGAT3
PC NP
PC NP
PC NP
FUT3
B3GNT3
B4GALNT3
FUT6
MGAT3
GCNT3
Figure S2: Data obtained for the selective glycoTs in pancreatic cancer and normal pancreas using the gepia (A – F) and mipanda (G – L) datasets.

## Slide 3
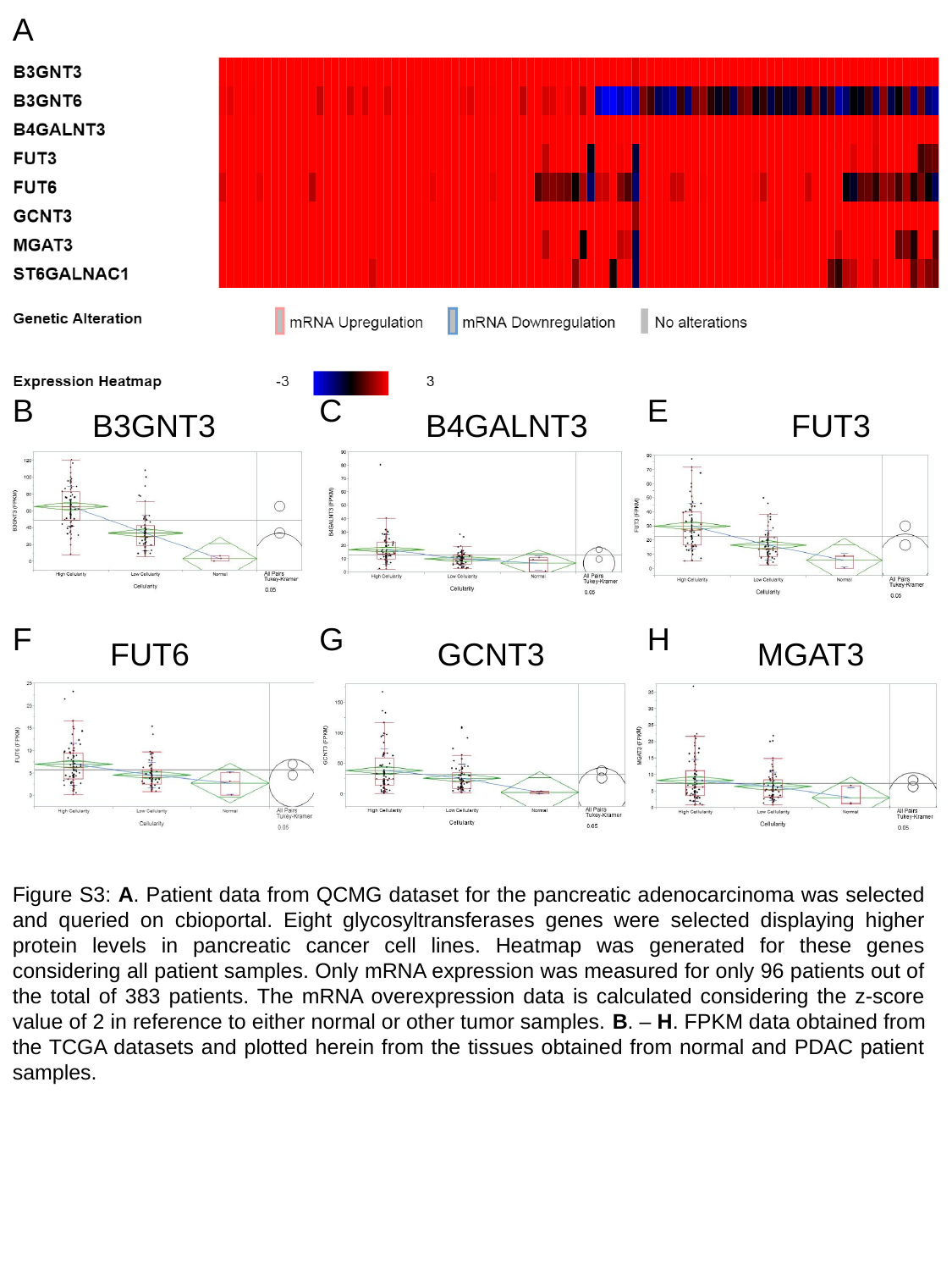

A
B		 C			E
F		 G			H
 B3GNT3	 B4GALNT3 FUT3
 FUT6 GCNT3 MGAT3
Figure S3: A. Patient data from QCMG dataset for the pancreatic adenocarcinoma was selected and queried on cbioportal. Eight glycosyltransferases genes were selected displaying higher protein levels in pancreatic cancer cell lines. Heatmap was generated for these genes considering all patient samples. Only mRNA expression was measured for only 96 patients out of the total of 383 patients. The mRNA overexpression data is calculated considering the z-score value of 2 in reference to either normal or other tumor samples. B. – H. FPKM data obtained from the TCGA datasets and plotted herein from the tissues obtained from normal and PDAC patient samples.

## Slide 4
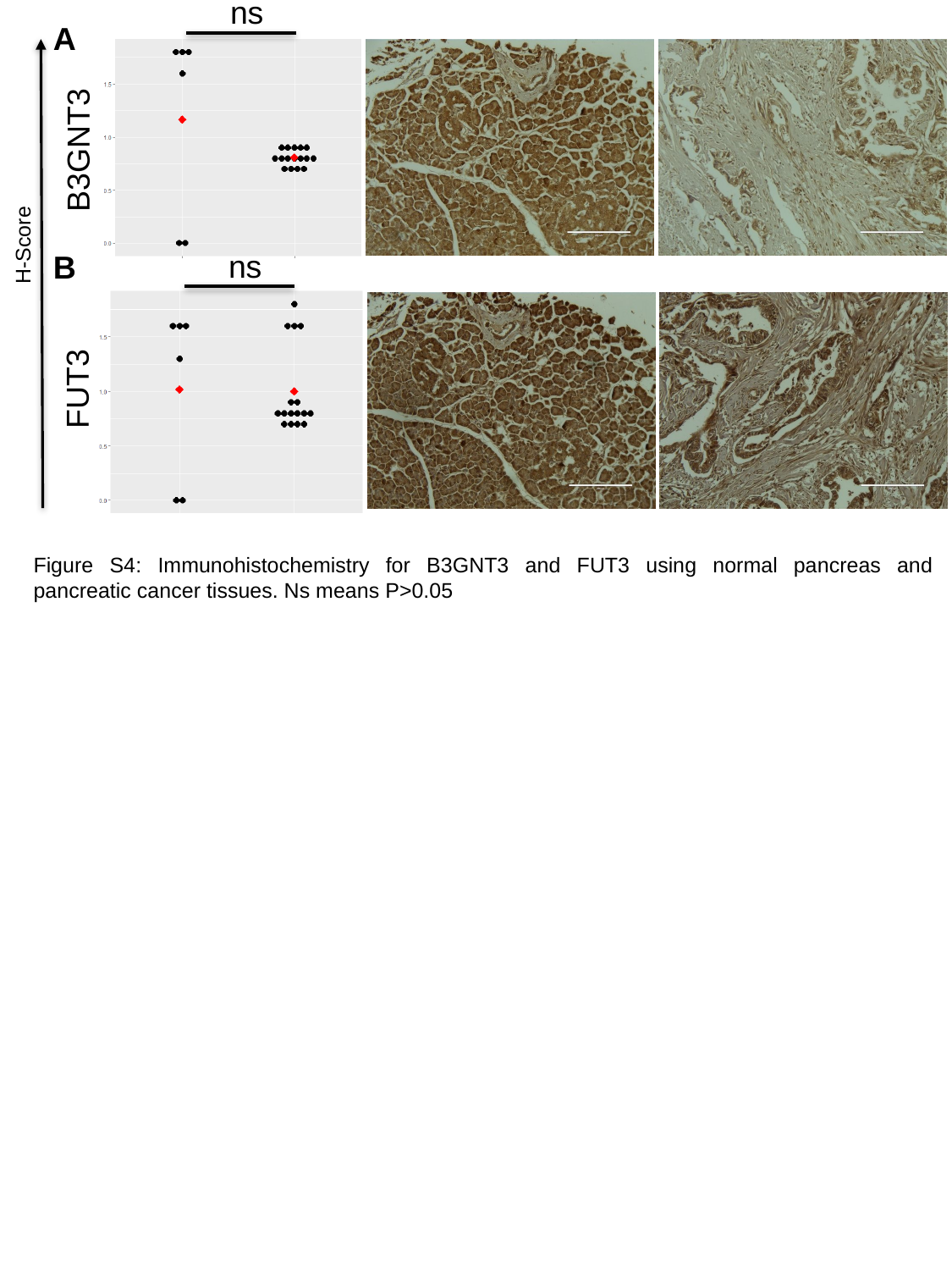

ns
A
B
B3GNT3
H-Score
ns
FUT3
Figure S4: Immunohistochemistry for B3GNT3 and FUT3 using normal pancreas and pancreatic cancer tissues. Ns means P>0.05

## Slide 5
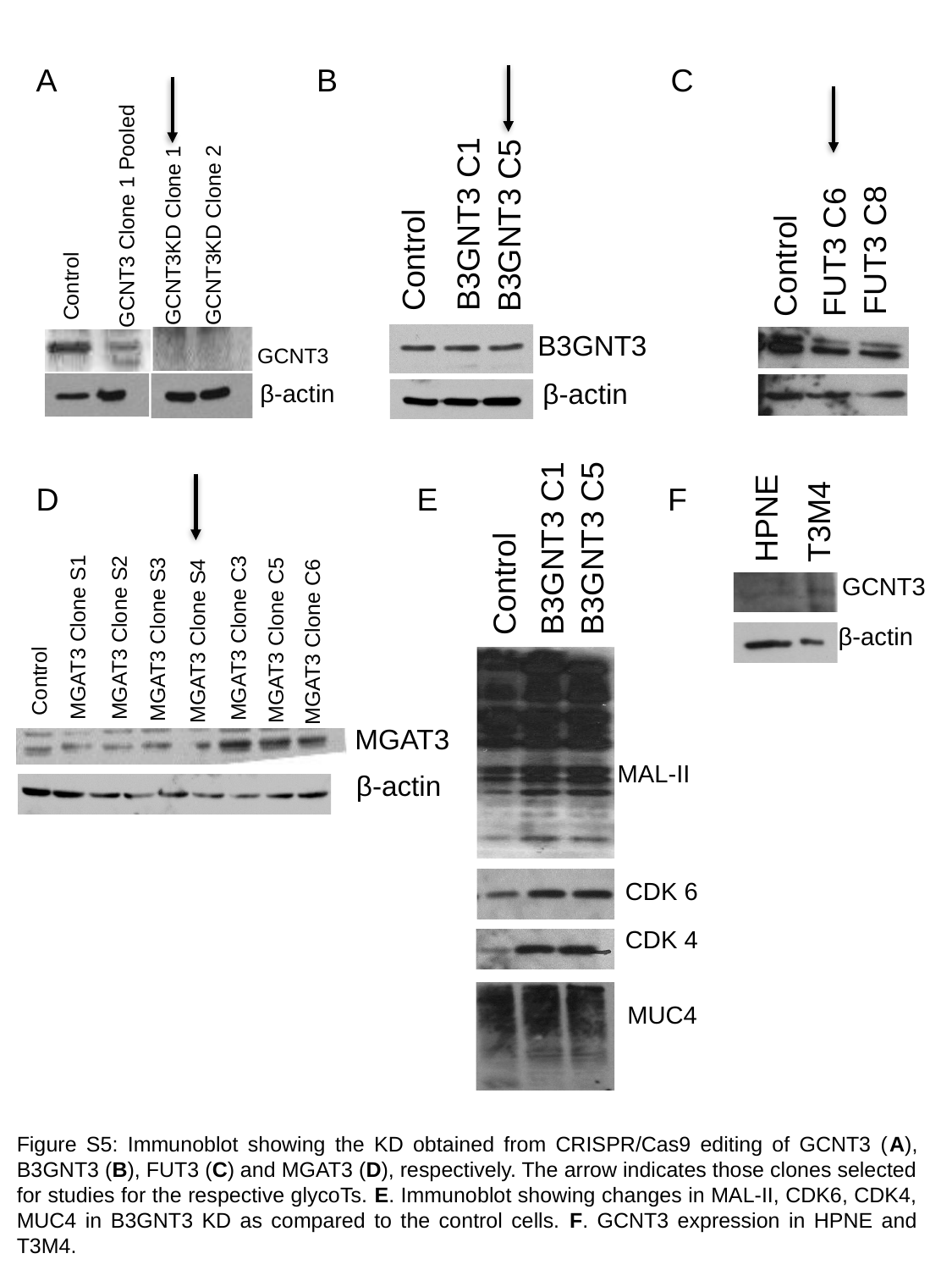

A		 B			C
D			E	 F
B3GNT3 C1
GCNT3 Clone 1 Pooled
B3GNT3 C5
GCNT3KD Clone 2
GCNT3KD Clone 1
Control
FUT3 C8
FUT3 C6
Control
Control
B3GNT3
GCNT3
β-actin
β-actin
HPNE
T3M4
Control
B3GNT3 C1
B3GNT3 C5
GCNT3
MGAT3 Clone S1
MGAT3 Clone S2
β-actin
MGAT3 Clone C3
MGAT3 Clone S3
MGAT3 Clone S4
MGAT3 Clone C5
Control
MGAT3 Clone C6
MGAT3
MAL-II
β-actin
CDK 6
CDK 4
MUC4
Figure S5: Immunoblot showing the KD obtained from CRISPR/Cas9 editing of GCNT3 (A), B3GNT3 (B), FUT3 (C) and MGAT3 (D), respectively. The arrow indicates those clones selected for studies for the respective glycoTs. E. Immunoblot showing changes in MAL-II, CDK6, CDK4, MUC4 in B3GNT3 KD as compared to the control cells. F. GCNT3 expression in HPNE and T3M4.

## Slide 6
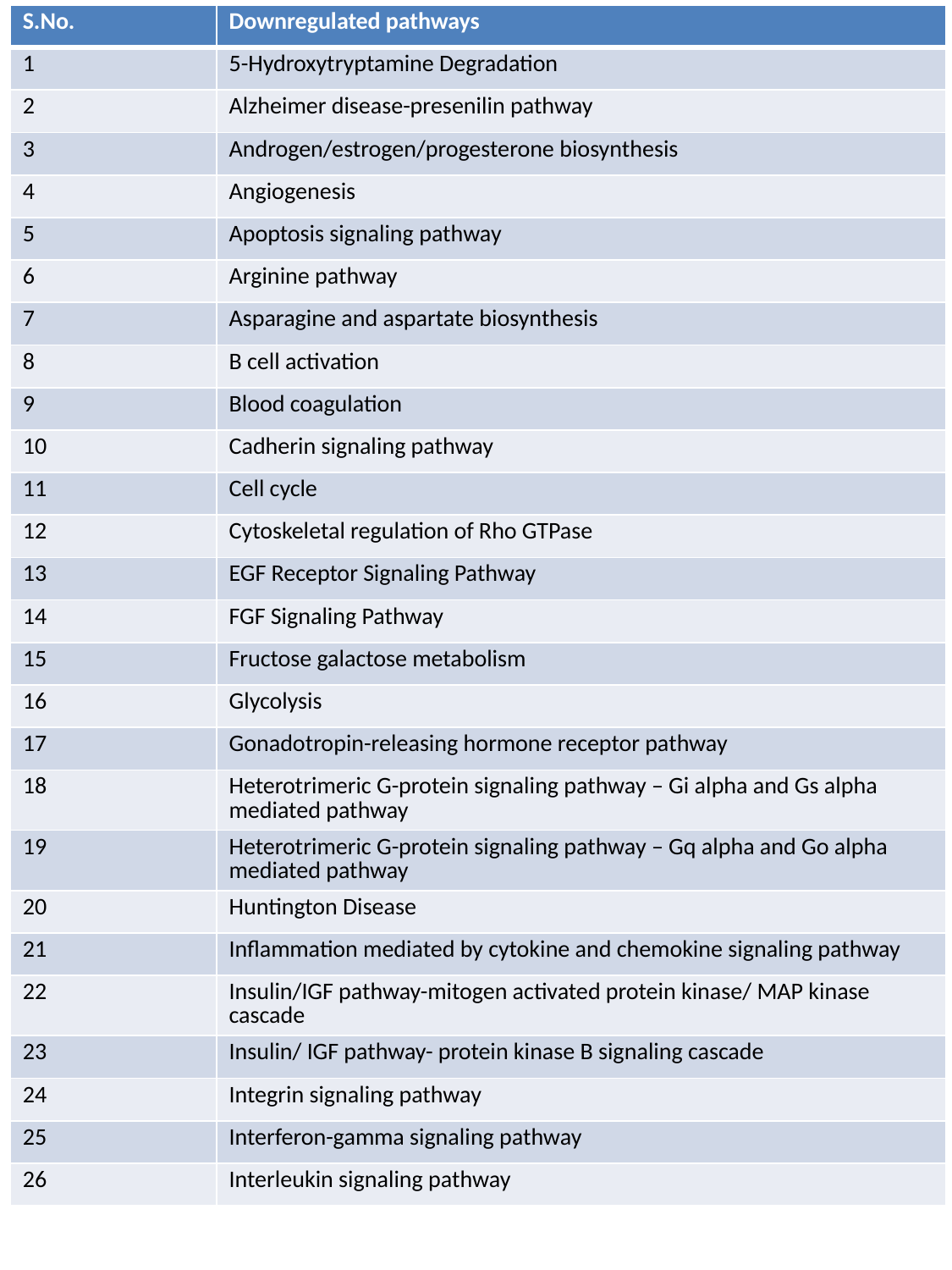

| S.No. | Downregulated pathways |
| --- | --- |
| 1 | 5-Hydroxytryptamine Degradation |
| 2 | Alzheimer disease-presenilin pathway |
| 3 | Androgen/estrogen/progesterone biosynthesis |
| 4 | Angiogenesis |
| 5 | Apoptosis signaling pathway |
| 6 | Arginine pathway |
| 7 | Asparagine and aspartate biosynthesis |
| 8 | B cell activation |
| 9 | Blood coagulation |
| 10 | Cadherin signaling pathway |
| 11 | Cell cycle |
| 12 | Cytoskeletal regulation of Rho GTPase |
| 13 | EGF Receptor Signaling Pathway |
| 14 | FGF Signaling Pathway |
| 15 | Fructose galactose metabolism |
| 16 | Glycolysis |
| 17 | Gonadotropin-releasing hormone receptor pathway |
| 18 | Heterotrimeric G-protein signaling pathway – Gi alpha and Gs alpha mediated pathway |
| 19 | Heterotrimeric G-protein signaling pathway – Gq alpha and Go alpha mediated pathway |
| 20 | Huntington Disease |
| 21 | Inflammation mediated by cytokine and chemokine signaling pathway |
| 22 | Insulin/IGF pathway-mitogen activated protein kinase/ MAP kinase cascade |
| 23 | Insulin/ IGF pathway- protein kinase B signaling cascade |
| 24 | Integrin signaling pathway |
| 25 | Interferon-gamma signaling pathway |
| 26 | Interleukin signaling pathway |

## Slide 7
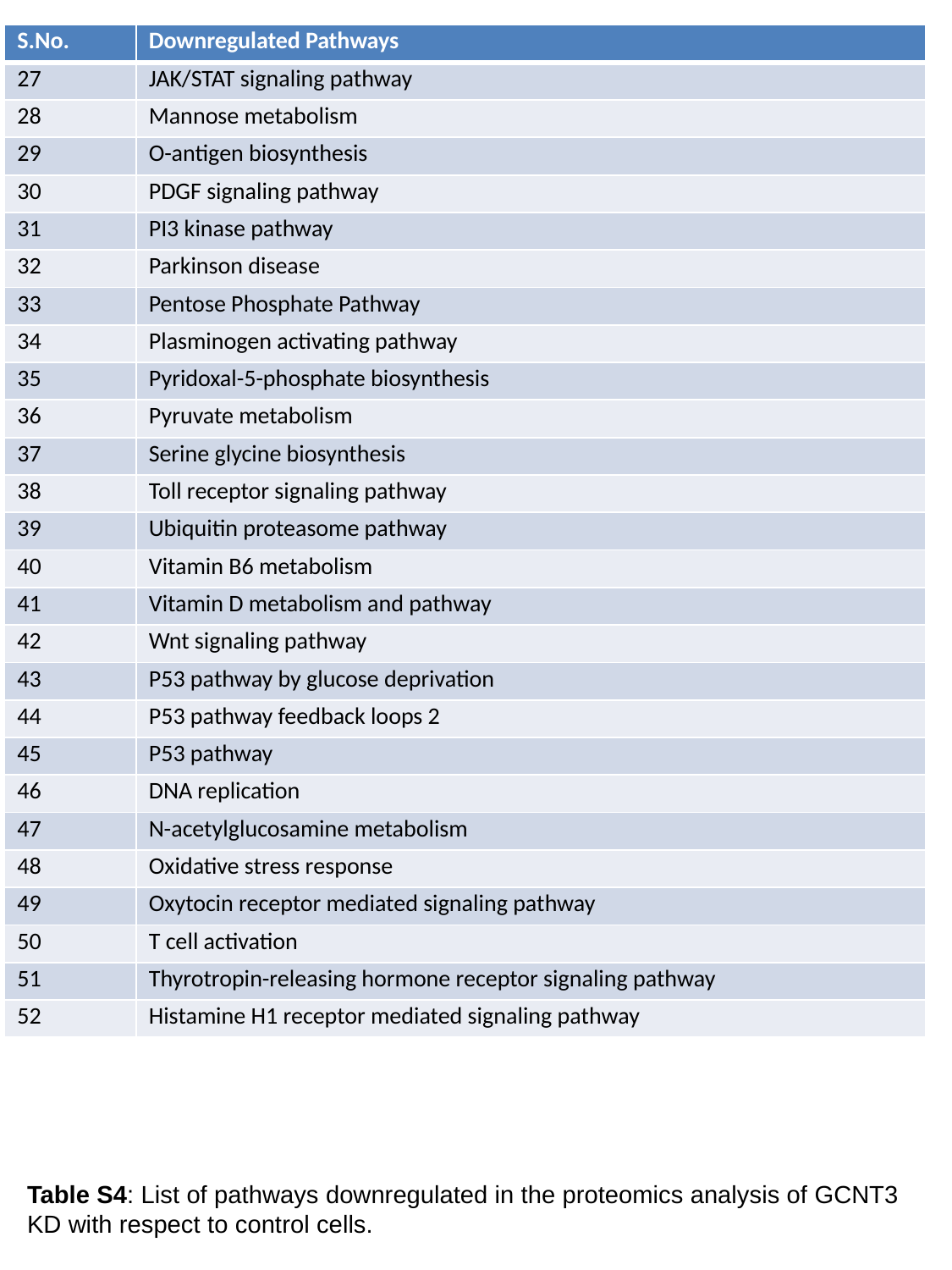

| S.No. | Downregulated Pathways |
| --- | --- |
| 27 | JAK/STAT signaling pathway |
| 28 | Mannose metabolism |
| 29 | O-antigen biosynthesis |
| 30 | PDGF signaling pathway |
| 31 | PI3 kinase pathway |
| 32 | Parkinson disease |
| 33 | Pentose Phosphate Pathway |
| 34 | Plasminogen activating pathway |
| 35 | Pyridoxal-5-phosphate biosynthesis |
| 36 | Pyruvate metabolism |
| 37 | Serine glycine biosynthesis |
| 38 | Toll receptor signaling pathway |
| 39 | Ubiquitin proteasome pathway |
| 40 | Vitamin B6 metabolism |
| 41 | Vitamin D metabolism and pathway |
| 42 | Wnt signaling pathway |
| 43 | P53 pathway by glucose deprivation |
| 44 | P53 pathway feedback loops 2 |
| 45 | P53 pathway |
| 46 | DNA replication |
| 47 | N-acetylglucosamine metabolism |
| 48 | Oxidative stress response |
| 49 | Oxytocin receptor mediated signaling pathway |
| 50 | T cell activation |
| 51 | Thyrotropin-releasing hormone receptor signaling pathway |
| 52 | Histamine H1 receptor mediated signaling pathway |
Table S4: List of pathways downregulated in the proteomics analysis of GCNT3 KD with respect to control cells.
