# Supplementary material for: Global analysis of human glycosyltransferases reveals novel targets for pancreatic cancer pathogenesis
Source: Br J Cancer. 2020 Mar 19;122(11):1661–72. doi: 10.1038/s41416-020-0772-3 (PMC7251111; doi:10.1038/s41416-020-0772-3)
Supplement: Supplementary file 2 — Supplemental document [file 41416_2020_772_MOESM2_ESM.docx]

| Table SI: List of human primer used for screening the transcriptional expression of glycosyltransferases. | | | |
| --- | --- | --- | --- |
| **S.No.** | **Gene Name** | **Forward Primer** | **Reverse Primer** |
| 1 | A4GNT | TTTCCTCTTCCCTTTGGATATGA | CGCTGGCGTTGATTTGATT |
| 2 | ABO | GCACTTCGACCTATGATCCTTT | GGCTTCCTGGCATTAGACTT |
| 3 | ALG1L | AGGTGCGGGATATAATCTTGTG | AGGTCCAGAGGTGTCTCTTTA |
| 4 | B3GALT5 | ACCCTGACTAATACACCTGGA | CCAAACACATCTAGACCACCA |
| 5 | B3GNT3 | CAAGTACTATGTGCCAGAGGTG | GAAGCGGGACAGCAAGAA |
| 6 | B3GNT5 | GTGGTGCCCCTCCCATTAG | GCTCCGGCTGTGTAGTCAGG |
| 7 | B3GNT6 | TCAAGAGAACTGAACCACAGG | AGAAGAAATAGACAGGAGCCATC |
| 8 | B3GNT7 | TGTTCCAACGCAGTCTCAC | GCCACATCTTTCGGGTTCT |
| 9 | B4GALNT2 | CGTGCGGAACGAACTCT | GAGCCGATTCCCACACTC |
| 10 | B4GALNT3 | TGTTGAGATGGCACTGAAGAG | TGGAGGTCACAGAGGAAGATG |
| 11 | B4GALT4 | CTGACTTTCCACCTTTCCTACA | AATCTCTTGAATGGCACCCA |
| 12 | C1GALT1 | CATCCCTTTGTGCCAGAACACC | GCAAGATCAGAGCAGCAACCAG |
| 13 | CHPF | AGGAGCGACCCATTGGA | TGTAGGTGGTGTCAGGCA |
| 14 | COLGALT2 | CACAGACCTCAGCTGTACGA | GGGCTCGCAGACAGTAGT |
| 15 | FUT2 | GATTCAAGCCATGTGGGAGTT | CGGCCTATTGCATTGATCGT |
| 16 | FUT3 | TAGGAGAGGCTGCCATATATCC | CAATTACTGCTTTGCACCCTTG |
| 17 | FUT6 | TGTGGAACCCGCTTTGG | ATTTGGAGACTCAGGTCATGC |
| 18 | FUT9 | CAAGGATTACATCACGGAAAAGC | TGGTCCCAGAACAACAGGTACA |
| 19 | GALNT12 | TGGTCAACTCTCCTTCGGA | AGGTGCTCTCTATCACTGTAGTC |
| 20 | GALNT3 | AAAGCGTTGGTCAGCCTCTA | AACGAGACCTTGAGCAGCAT |
| 21 | GALNT5 | GAGAAACGGCAGTCTCAATCT | CACCGAACAGTTCAACATATCAC |
| 22 | GCNT1 | TGCTTCCTCCACTCGAAACA | TGTCTTGTGCCCACTCCATC |
| 23 | GCNT3 | TACTTGTGACCTGCCCTTTAC | GTTTCCCTTCAGCACCTACA |
| 24 | HAS1 | AGCCTCTTCGCGTACCT | GATGGTCAGCGCCACAC |
| 25 | HAS2 | CCATTGAACCAGAGACTTGAAAC | TGTGGAAGACTCAGCAGAAC |
| 26 | HAS3 | CACTGCGGAATTCAAAGCTAAG | GAAAGAGGCGCTGAAGAGAA |
| 27 | LFNG | CCTCTTCCACTCCCACCT | CGGCGTTCCGCTTGTTT |
| 28 | MGAT3 | GAGTCCAACTTCACGGCTTAT | GATGTACTCGAAGGTGCCATT |
| 29 | PYGB | CCCGCGACTACTTCTTCG | ATCGTGTTCTGCAGCGT |
| 30 | ST3Gal-I | CAGAGATGGACGGTCACT | CAACTGTGGTTTCTGACG |
| 31 | ST6GALNAC1 | AGGCACAGACCCCAGGAAG | TGAAGCCATAAGCACTCACC |
| 32 | ST6GALNAC2 | CTTTGCCCTGTACTTCTCG | CAGCACTGGAATGGAGAGA |
| 33 | ST8SIA2 | GAGATCGAAGAAGAAATCGGGAA | GGTGATGAGGAGCCGTTTATTA |
| 34 | UGT1A6 | CATTCCTAACTGCTCCTCAGAC | GCATCAAACTTGCTCTCCTTAAA |
| 35 | UGT1A7 | CAGTGAAGACTTACTCAACCTCATAC | GCAATGGTGCCGTCCAG |
| 36 | UGT1A8 | TGTATCATAGCAGCTTAGAATCCC | TCAGCAGCAGAGAAACACATAG |
| 37 | UGT2B4 | CTGTGTGGCAACTGTGATATTC | CTTCCAGCCTCAGACGTAAT |
| 38 | UGT2B7 | TTTCACAAGTACAGGAAATCATGTCAAT | CAGCAGCTCACTACAGGGAAAAAT |
| 39 | UGT8 | TGAGCCAAGTGCGGAAG | CTCTCTGGGTTCACAGTTGATAG |

| Table SII: List of mouse primers used for screening the transcriptional expression of glycosyl transferases from mouse tissues. | | | |
| --- | --- | --- | --- |
| **S.No.** | **Gene Name** | **Forward Primer** | **Reverse Primer** |
| 1 | mAbo | ATCTCAGAGGAAGACCGAAATG | CTTAGGAACAGGTAGCCAAAGA |
| 2 | mB3galt5 | GGAAACGGCAGAGGACATT | CGGAAATAACTTCTCCAGTACGA |
| 3 | mB3gnt3 | CGGTGCCGAGCTAATCTATC | CAGTGGCGGTAGAACAAGAA |
| 4 | mB3gnt6 | GCCAGATTCTCCTCTCTCAAAC | CCACTGATTCAGCACCACTAA |
| 5 | mB3gnt7 | CTCAGGATGTTGGCGATCTT | CCCAGGTAGGTTAACCCTTTG |
| 6 | mB4galnt2 | TTTGTCCTCATGGTAGGACTTG | GGAAACTTTGGCTTGAAGGTG |
| 7 | mB4galnt3 | TGGGACTCTGGACTCTGTATC | GCTGCCATACCTGTGGTT |
| 8 | mC1galt1 | GCTTTGTCTGGAGCTGGT | GAATGTATTTCTGGCTGGTTCAC |
| 9 | mFut2 | CGGCTAAGGTACATCTATCAGC | GGTGGAAGTCACAAAGACAAAG |
| 10 | mFut9 | TCTGGAGCCCTGGATCTT | CTAGTAGCGTAGCCGGAGT |
| 11 | mGalnt3 | CGGCAAACCTATGCTAAAGAGTATAA | GCCATTCTGTCATTAAATGCTTGTT |
| 12 | mGalnt5 | GGAGCGGGCACAAATACT | AGCAGGAAATAGCTGTCCAAG |
| 13 | mGcnt3 | ATCTATCTGGGAGGTACGAAGG | CGTGAGTGATGTGGAGTGAAG |
| 14 | mMgat3 | GACGATGGGATGAAGATGAGAC | AAAGAAGTGCAGGAAGGATATGA |
| 15 | mSt6galnac1 | GGTGCCAGTTCATATGGAAGA | AGAGGTTGTCTCCTTGTTGTG |
| 16 | mSt6galnac2 | CGGAAACCTCTGCCAGTAAA | TCGTGTGTTAATCAGCTTGGA |
| 17 | mUgt1a6a | ACCGACTTCCAGATTTCTTACTC | GCAAGCCATCCTGTCAGATA |

| Table SIII: List of sgRNA used for CRISPR/Cas9 editing of glycosyltransferases in PC cell lines. | | |
| --- | --- | --- |
| **S.No.** | **Gene Name** | **sgRNA** |
| 1 | B3GNT3 | GGATGAAGTATCTCCGGCAC |
| 2 | B4GALNT3 | ACGTGGCGGAGATGCGACTT |
| 3 | FUT3 | TGAACTTAGGTCTTGGCGTC |
| 4 | FUT6 | GAACACCCTATGGCTGACCG |
| 5 | GCNT3 | TGAAGACTGGCGGTGTTCCG |
| 6 | MGAT3 | GGACGTGCGCTTCCACGAGC |

Antibodies corresponding to B3GNT3, B4GALNT3, FUT3, FUT6, GCNT3, MGAT3, SOX9 were obtained from (Abcam, Cambridge, MA, USA); SOX2, P-glycoprotein (Santa Cruz Biotechnology, Dallas, TX, USA); ZO-1, Zeb-1, Snail, CD44 (Cell Signaling Technology, Danvers, MA, USA); E-cadherin, N-cadherin; MUC4 (Developed Inhouse); β-catenin (Sigma)

**KPC mouse model and animal euthanasia**

Genetically-engineered mouse (GEM) models offer early-stage PC samples, which are very difficult to get from human patients. These models also mimic the histopathology of human PC progression. Currently, we have the most widely used mouse models for PC KPC models, which express either constitutively activated KrasG12D and Trp53R172H (KPC) together in the pancreas. We have established a widely used KPC model, which consists of triple transgenic animals (KrasG12D; Trp53R172H/+; Pdx-1-Cre) obtained by crossing LSL-KrasG12D with LSL-Trp53R172H/+, transgenic animals. Subsequently, the F1 progeny positive for p53 and KrasG12D is further crossed with Pdx-1-Cre to generate triple transgenic animals (KrasG12D; Trp53R172H/+; Pdx-1-Cre/KPC model). The KPC model involves targeted expression of an endogenous KrasG12D allele and Trp53R172H/+ in murine pancreatic progenitor cells and closely recapitulates many of the genetic alterations, histopathology and metastatic features of human PC, including an intense stromal response. The KPC mice develop preneoplastic lesions at 5 weeks after birth and invasive tumor at 25 weeks of age. We have collected the pancreas of KPC at 5th and 25th week and isolated RNA to validate the GTs.

**Method of Euthanasia**

**Method of Euthanasia:** Euthanasia and premature euthanasia were performed by using CO_2_ asphyxiation followed by cervical dislocation.

**Criteria for Euthanasia:** Animals were euthanized at the experimental end points, defined as that period of time after the initiation of tumors when obvious signs of autoimmune disease appear (the obvious steps of autoimmune disease include inflammation or evidence of inflammation in any of the affected organs). Animals maintained as breeding stock were euthanized by CO_2_ followed by cervical dislocation when they were deemed to show no reproductive capacity or after 18 months of age, whichever occured first.

**Criteria for Death:** Animals observed to show no signs of breathing and no heartbeat for 5 minutes are deemed dead.

**Criteria for Premature Euthanasia:** Animals euthanized at the earliest possible endpoint when they were in pain or if they developed an infection.

**Mass Spectrometry based proteomics**:

500 - 1000 ug of protein per sample was taken and detergent was removed by chloroform/methanol extraction, and the protein pellet was re-suspended in 100 mM ammonium bicarbonate and digested with MS-grade trypsin (Pierce) overnight at 37°C with. Peptides cleaned with PepClean C18 spin columns (Thermo) were re-suspended in 2% acetonitrile (ACN) and 0.1% formic acid (FA) and 500 ng of each sample was loaded onto trap column Acclaim PepMap 100 75µm x 2 cm C18 LC Columns (Thermo Scientific™) at flow rate of 4 µl/min then separated with a Thermo RSLC Ultimate 3000 (Thermo Scientific™) on a Thermo Easy-Spray PepMap RSLC C18 75µm x 50cm C-18 2 μm column (Thermo Scientific™) with a step gradient of 4–25% solvent B (0.1% FA in 80 % ACN) from 10-130 min and 25–45% solvent B for 130–145 min at 300 nL/min and 50^o^C with a 180 min total run time. Eluted peptides were analyzed by a Thermo Orbitrap Fusion Lumos Tribrid (Thermo Scientific™) mass spectrometer in a data dependent acquisition mode. A survey full scan MS (from m/z 350–1800) was acquired in the Orbitrap with a resolution of 120,000. The AGC target for MS1 was set as 4 × 10^5^ and ion filling time set as 100 ms. The most intense ions with charge state 2-6 were isolated in 3 s cycle and fragmented using HCD fragmentation with 35 % normalized collision energy and detected at a mass resolution of 30,000 at 200 m/z. The AGC target for MS/MS was set as 5 × 10^4^ and ion filling time set 60 ms dynamic exclusion was set for 30 s with a 10 ppm mass window. Protein identification was performed by searching MS/MS data against the swiss-prot human protein database downloaded on Feb 13, 2019 using sequest search engine. The search was set up for full tryptic peptides with a maximum of two missed cleavage sites. Acetylation of protein N-terminus and oxidized methionine were included as variable modifications and carbamidomethylation of cysteine was set as fixed modification. The precursor mass tolerance threshold was set 10 ppm for and maximum fragment mass error was 0.02 Da. The significance threshold of the ion score was calculated based on a false discovery rate of ≤ 1%. Qualitative analysis was performed using Proteome Discoverer 2.3.
